# Single-cell epigenomics of colorectal cancer

**DOI:** 10.1038/s12276-026-01855-4

**Published:** 2026-10-02

**Authors:** Dain Kang, Jun Ho Lee, Inkyung Jung

**Affiliations:** 1 Department of Biological Sciences, Korea Advanced Institute of Science and Technology, Daejeon, Republic of Korea; 2 Graduate School of Stem Cell and Regenerative Biology, Korea Advanced Institute of Science and Technology, Daejeon, Republic of Korea; 3 KAIST InnoCORE AI-CRED Institute, Korea Advanced Institute of Science and Technology, Daejeon, Republic of Korea; 4 Department of AX, College of AI, Korea Advanced Institute of Science and Technology, Daejeon, Republic of Korea

**Keywords:** Epigenetics, Colorectal cancer, Tumour heterogeneity

## Abstract

Colorectal cancer (CRC) exhibits remarkable phenotypic heterogeneity that cannot be fully explained by genetic alterations alone. Advances in single-nucleus assay for transposase-accessible chromatin sequencing have revealed chromatin accessibility as a central regulatory layer shaping tumor identity, cellular plasticity and therapeutic response. In this Review, we synthesize recent single-cell epigenomic studies to outline an emerging regulatory principle for CRC. From an epigenetic perspective, CRC can be conceptualized as two distinct epigenomic states, intrinsic consensus molecular subtype (iCMS)2 and iCMS3, defined by divergent *cis*-regulatory element activities across distal enhancer and promoter landscapes. Tumor progression is accompanied by stepwise regulatory rewiring, encompassing erosion of differentiation programs in adenomas, regulatory bifurcation during malignant transformation, oncofetal reprogramming associated with therapy resistance and organotropic adaptation during metastasis. We further discuss how stromal and immune compartments undergo dynamic epigenomic remodeling, converging toward a concept of iCMS-defined epigenomic ecosystems, and how genomic alterations engage in bidirectional crosstalk with chromatin states to generate intratumoral heterogeneity. Finally, we highlight translational applications including epigenetic–immune combination therapies, liquid biopsy-based monitoring, spatial epigenomics and artificial intelligence-driven modeling.

## Tumor identity shaped by epigenomic regulatory landscapes

Colorectal cancer (CRC) is a leading cause of cancer-related deaths, characterized by genetic mutations and phenotypic plasticity. Recent studies have highlighted the role of nongenetic mechanisms, particularly epigenomic plasticity, in CRC progression. Here we explore how single-cell chromatin accessibility profiling has advanced our understanding of CRC by revealing distinct regulatory states and dynamic chromatin remodeling. The authors synthesized recent findings from single-cell epigenomics, highlighting iCMS2 and iCMS3 as two main epigenomic states with distinct *cis*-regulatory landscapes. They further integrated transcription factor-regulated phenotypic plasticity and oncofetal reprogramming in CRC, both contributing to therapeutic resistance. These findings suggest that chromatin accessibility patterns can predict tumor lineage and therapeutic vulnerabilities. This review proposes that targeting epigenomic states could enhance precision oncology, with future research focusing on integrating spatial epigenomics and artificial intelligence-driven models to further elucidate CRC’s regulatory architecture. This summary was initially drafted using artificial intelligence, then revised and fact-checked by the author.

## Introduction

Colorectal cancer (CRC) remains one of the most prevalent and lethal malignancies worldwide, accounting for nearly 2 million new cases and close to 1 million deaths annually^1^. Despite advances in early detection and therapeutic intervention, CRC continues to exhibit high rates of recurrence, metastasis and therapeutic resistance. Traditionally, CRC initiation and progression have been explained by a stepwise genetic model involving the sequential accumulation of driver mutations in genes such as *APC, KRAS, SMAD4* and *TP53*^2^. In brief, *APC* mutation is a predominant driver mutation in approximately 80% of patients with CRC^3^, suggesting that it enables tumor cells to outcompete neighboring wild-type intestinal stem cells at the initiation of mouse intestinal tumor^4^. However, *KRAS* and *BRAF*, which are frequently mutated (44% and 8.6%, respectively) in microsatellite-stable CRC^5^, are known to promote an epithelial identity skewed toward a regenerative/fetal-like state^6^.

A recent multiregional whole-genome sequencing study using human organoids has revealed that the phenotypic plasticity occurs in the absence of additional driver mutations^7^. Extending this observation, genetically identical cancer cells can give rise to divergent cellular states^8^, challenging genotype-based predictions of disease progression and therapeutic response. Interestingly, targeted MAPK inhibition induces epithelial plasticity by shifting toward a WNT-associated canonical stem cell state^6^, thereby driving therapeutic resistance. These observations are consistent with the remarkable plasticity of gene expression in CRC^9^. Taken together, although stepwise accumulation of genetic mutations in CRC is well established, nongenetic mechanisms are increasingly implicated as major contributors to CRC phenotypic plasticity.

To characterize molecular phenotypic heterogeneity, single-cell transcriptomic (single-cell RNA sequencing, scRNA-seq) technologies have been widely applied to CRC, enabling systematic dissection of malignant epithelial populations, cancer-associated fibroblasts (CAFs), endothelial cells and diverse immune subsets within the tumor microenvironment (TME)^10–12^. Specifically, large-scale integrative analyses of scRNA-seq datasets have revealed functional divergence of CAFs into inflammatory CAFs and myofibroblastic CAFs, which contribute to immune evasion and extracellular matrix remodeling, respectively^10,13^. In addition, functional diversification of stromal and immune compartments including exhausted T cells, immunosuppressive macrophages and tertiary lymphoid structure-associated B cells, correlate with patient prognosis and therapeutic response^10,11^. However, scRNA-seq primarily captures gene expression outputs rather than the regulatory mechanisms that establish these cellular states, limiting our ability to decode the epigenetic basis of phenotypic plasticity in CRC. Thus, the transcriptome alone, even at unprecedented resolution, is insufficient for decoding the underlying heterogeneous and transient properties driving CRC progression.

Cellular heterogeneity in CRC is therefore more fundamentally rooted in the organization and plasticity of gene regulatory networks. Earlier bulk epigenomic studies revealed that CRC is characterized by extensive reprogramming of chromatin states, exemplified by the CpG island methylator phenotype (CIMP)^14^ and widespread promoter hypermethylation that arise early during adenoma formation and continue to evolve during tumor progression^15^. More recent multi-omic analyses, including those from the European Prospective Investigation into Cancer and Nutrition (EPIC) consortium, have demonstrated that epigenomic plasticity rather than clonal genetic mutation is a dominant driver of intratumoral heterogeneity in CRC, enabling genetically identical cells to adopt divergent phenotypes under microenvironmental or therapeutic pressures^9,16^. These paradigms align with emerging models of ‘nongenetic cancer evolution’, in which chromatin accessibility and regulatory rewiring fuel phenotypic diversity and adaptive resistance^17^.

Recent advances in single-cell epigenomic technologies have begun to resolve the regulatory architecture underlying CRC cellular states. Single-cell and single-nucleus chromatin accessibility profiling approaches, including single-cell assay for transposase-accessible chromatin sequencing (scATAC-seq)^18,19^ and multimodal platforms^20,21^, enable direct interrogation of *cis*-regulatory landscapes at cellular resolution. Among these, single-nucleus (sn)ATAC-seq has emerged as a particularly robust and widely adopted method owing to its compatibility with frozen and archived human CRC specimens, high reproducibility, efficient detection of accessible chromatin regions and seamless integration with single-cell transcriptomic data^20,21^. By capturing the regulatory potential of individual cells, snATAC-seq provides a mechanistic and upstream readout of cellular identity, lineage competence and phenotypic plasticity beyond what transcriptional profiling alone can offer.

**Fig. 1:**
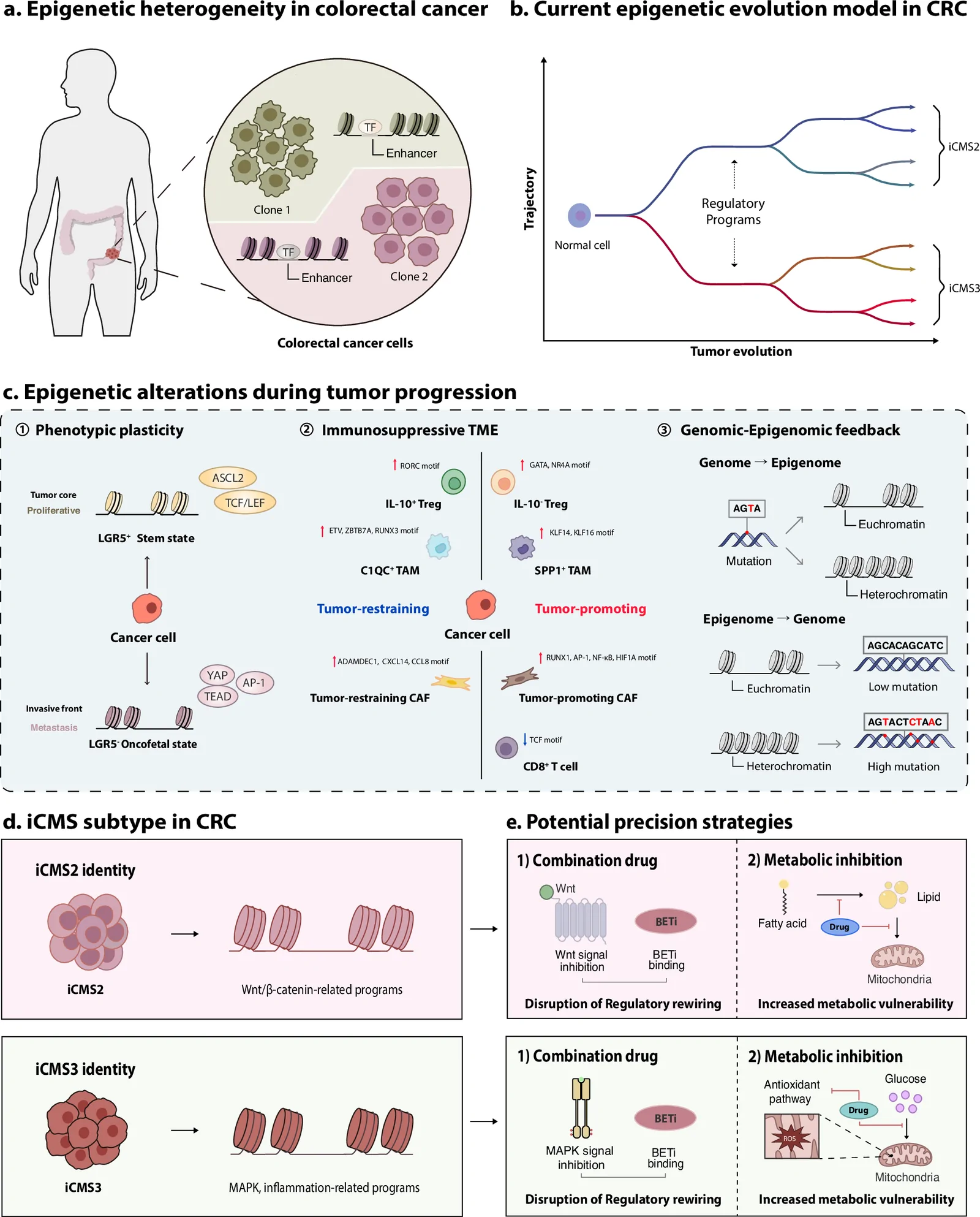
Integrating snATAC-seq to unravel epigenetic heterogeneity in CRC: from current insights to translational applications. **a** Illustration of epigenetic heterogeneity within the same patient with CRC, influenced by clonal diversification. Each tumor clone harbors distinct chromatin accessibility landscapes, enabling the recruitment of different transcription factors (TFs) to regulatory elements such as enhancers. **b** A recent framework proposing dichotomous iCMS states^27^, which was originally defined through transcriptomic analysis^56^. These two iCMS states are governed by a different landscape of chromatin accessibility. **c** Illustration of key epigenetic alterations that occur during colorectal tumorigenesis, encompassing (1) phenotypic plasticity ranging from LGR5^+^ stem-like to *LGR5*^−^ oncofetal state, mediated by transcription factors; (2) dynamic chromatin remodeling within the TME, shaping tumor-restraining or tumor-promoting states during CRC progression; and (3) genomic–epigenomic crosstalk influencing tumor cell evolution. **d** Shown are different regulatory programs and signaling dependencies of two epigenomic states, iCMS2 and iCMS3, in CRC. **e** Molecular stratification of iCMS2 and iCMS3 provides a future framework for precision oncology, potentially enabling combination therapies tailored to state-specific signaling pathways and metabolic vulnerabilities.

With the rapid accumulation of single-cell epigenome studies in CRC^11,22–31^ (Table 1), there is a growing need to systematically organize these findings to outline an emerging regulatory principle. In this Review, we discuss how single-cell chromatin accessibility profiling has transformed our understanding of CRC (Fig. 1). We first examine how regulatory architecture defines CRC identity through distinct epigenomic states that reflect cellular identity^24,25,27^. We then explore how dynamic regulatory rewiring characterizes each regulatory stage from adenoma to metastasis^24,27,30,32–36^. Next, we describe how microenvironmental populations undergo dynamic epigenetic reprogramming and suggest the concept of an ‘epigenomic ecosystem’ as a future direction^11,24,25,37–45^. We further discuss how genomic alterations reshape the epigenetic landscape^22,46–52^. Finally, we highlight emerging translational applications including epigenetic–immune targeting, liquid biopsy, spatial epigenomics and artificial intelligence (AI)-driven modeling^22,23,28,29,53–58^. Collectively, these insights establish single-cell epigenomics as both a mechanistic framework for understanding CRC evolution and a translational platform for precision oncology.

**Table 1:** Overview of sample types and publicly available snATAC-seq single-cell epigenome datasets in CRC.

Sample type	Paper	Samples analyzed in the study	scATAC-seq application in CRC	Integrated analysis data	Dataset accession	Alignment tools	Analysis tools
Clinical sample	Becker et al. (2022)^24^	Human CRC, polyp, normal tissue	8 FAP, 4 sporadic CRC, 3 healthy control tissues (normal colon, polyps, adenocarcinomas)	snRNA-seq	GSE201349 (https://www.ncbi.nlm.nih.gov/geo/query/acc.cgi?acc=GSE201349)	Cell Ranger	ArchR
	Liu et al. (2024)^27^	Paired human CRC tissues and adjacent normal tissues	28 CRC, 1 rectal adenoma tissues, matched adjacent normal tissues (23 MSS, 6 MSI-H)	n.a.	HRA000992 (GSA-Human) (https://ngdc.cncb.ac.cn/gsa-human/browse/HRA000992)	Cutadapt - hisat2 - picard - sinto	ArchR
	Sundaram et al. (2024)^22^	74 Tumor samples, healthy reference atlas	Human CRC, normal colon tissues	n.a.	https://gdc.cancer.gov/about-data/publications/TCGA-ATAC-Seq	Cell Ranger	ArchR
	Shanmugam et al. (2024)^26^	Human CRC, adjacent tissues, PDX, PDO, PDC	6 Human CRC tissues	TRAP assay (classification)	SCP2592 (Broad Institute) (https://singlecell.broadinstitute.org/single_cell/study/SCP2592/iron-fe3-dependent-reactivation-of-telomerase-drives-colorectal-cancer	Cell Ranger	Signac
	Yu et al. (2025)^25^	Paired human CRC tissues and adjacent normal tissues	9 Single-cell multi-omes	snRNA-seq	GSE294559 (https://www.ncbi.nlm.nih.gov/geo/query/acc.cgi?acc=GSE294559)	Cell Ranger	Signac
	Nguyen et al. and Lee et al. (2026)^31,78^	131 Human tumor samples, 3 healthy tissues, and peripheral blood	24 CRC and 9 adjacent nonmalignant tissues	snRNA-seq	GSE306459 (https://www.ncbi.nlm.nih.gov/geo/query/acc.cgi?acc=GSE306459)	Cell Ranger	Signac
Cell-line sample	Zhu et al. (2023)^46^	40 Human cancer cell lines, 2 human normal cell lines	11 CRC-related cell lines	scRNA-seq	CNP0004330 (CNSA, raw) (https://db.cngb.org/data_resources/project/CNP0004330) CNP0003658 (CNSA, processed) (https://db.cngb.org/data_resources/project/CNP0003658)	PISA	ArchR
	Hung et al. (2024)^52^	6 Human cell lines	COLO 320DM cell line	scRNA-seq (multi-ome), scCircle-seq	GSE159986 (https://www.ncbi.nlm.nih.gov/geo/query/acc.cgi?acc=GSE159986)	Cell Ranger	ArchR
	Ladaika et al. (2025)^102^	HT29 cell line, orthotopic xenograft mouse, human ascending colon organoid (normal, tumor)	HT29 cell line	scRNA-seq	GSE268957 (https://www.ncbi.nlm.nih.gov/geo/query/acc.cgi?acc=GSE268957)	Cell Ranger-ARC	Signac
Model organism sample	Li et al. (2023)^30^	Liver metastastic orthotopic PDX (Balb/c nude mice)	3 Paired primary and liver metastasis tumor tissues	n.a.	GSE200813 (https://www.ncbi.nlm.nih.gov/geo/query/acc.cgi?acc=GSE200813)	Cell Ranger	Signac
	Deng et al. (2024)^29^	Three CRC cell lines, MC38 mice tissues, PDO	MC38 Syngeneic mice tumor tissues	scRNA-seq	GSE197229 (https://www.ncbi.nlm.nih.gov/geo/query/acc.cgi?acc=GSE197229)	Cell Ranger	ArchR
Organoid sample	Mzoughi et al. (2025)^36^	Isogenic mouse organoid model (A, AK, AKS, AKSP), VAKSP tumoroid, PDO	WT, APC organoid, isogenic AKSP mouse tumoroid	scRNA-seq	GSE237954 (https://www.ncbi.nlm.nih.gov/geo/query/acc.cgi?acc=GSE237954)	Cell Ranger-ARC	Signac
	Yu et al. (2025)^25^	Paired human CRC & normal PDO	9 Single-cell multi-omes (snATAC-seq and snRNA-seq)	snRNA-seq	GSE294559 (https://www.ncbi.nlm.nih.gov/geo/query/acc.cgi?acc=GSE294559)	Cell Ranger	Signac

The term ‘integrated’ indicates that the analysis was used together with scATAC-seq.

Only the primary tool is captured.

*MSS* microsatellite stable, *MSI-H* microsatellite instability-high, *PDX* patient-derived xenograft, *PDO* patient-derived organoid, *PDC* patient-derived cell, FAP familial adenomatous polyposis, *scCircle-seq* single-cell Circle sequencing, *TRAP* Telomeric Repeat Amplification Protocol, *WT* wild-type, *A* Apc^null^, *AK* Apc^null^::Kras^G12D^, *AKS *Apc^null^::Kras^G12D^::Smad4^null^, *AKSP* Apc^null^::Kras^G12D^::Smad4^null^::Trp53^null, *VAKSP* Villin-Cre AKSP^

## Results

### Regulatory architecture specifies CRC identity

**Fig. 2:**
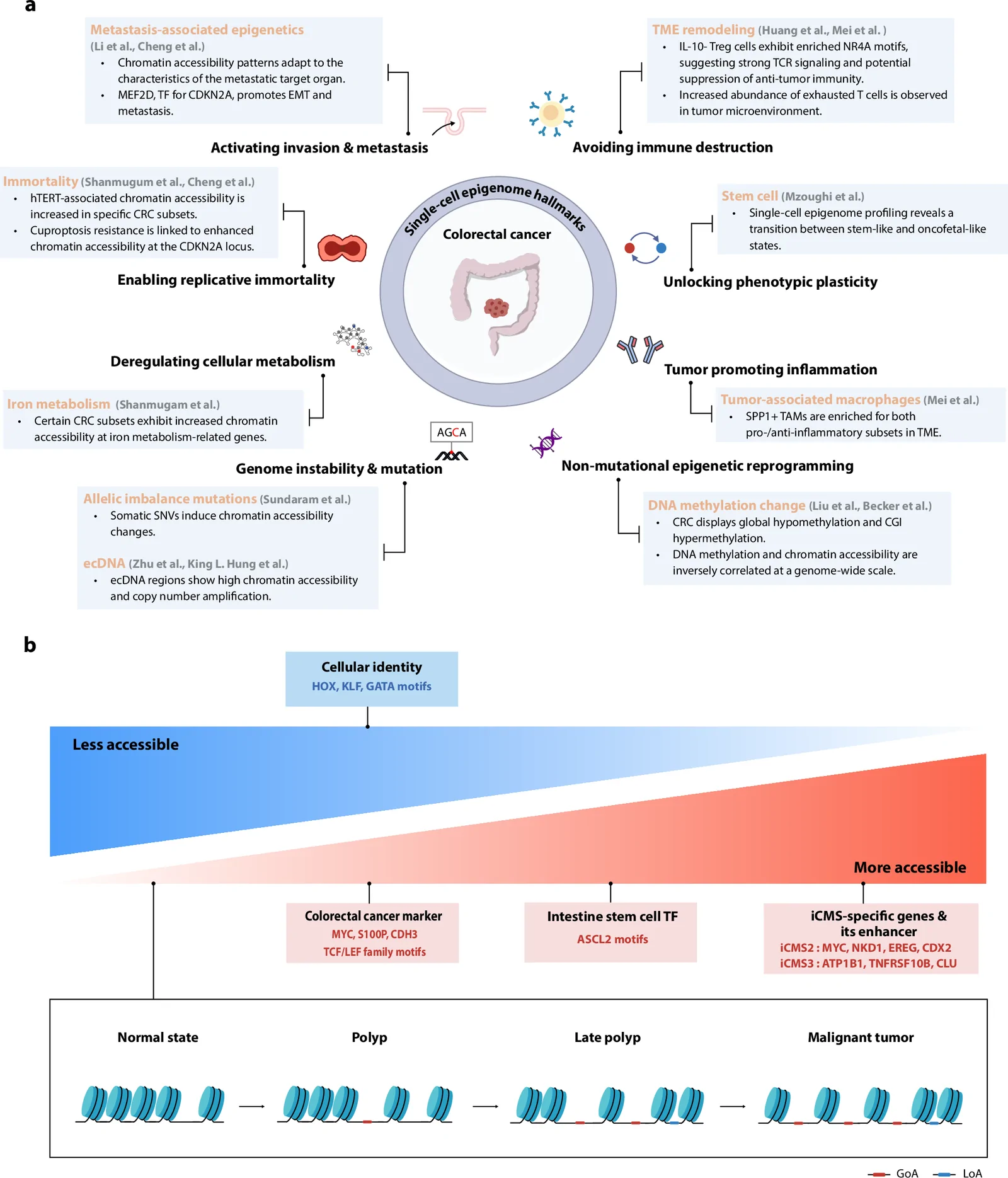
Single-cell epigenomic landscape of CRC: hallmarks and dynamic chromatin remodeling. **a** Overview of single-cell epigenomic studies of CRC organized according to the classical hallmarks of cancer. Chromatin accessibility profiling links regulatory programs to hallmark processes, including proliferative signaling, invasion and metastasis, immune evasion, inflammation, metabolic rewiring, and genome instability. **b** The schematic illustrates progressive regulatory changes along the normal tissue–polyp–malignant tumor continuum. Regions exhibiting gain of accessibility or loss of accessibility relative to normal tissue are highlighted. Representative transcription factor (TF) motifs and regulatory programs, including intestinal stem cell-associated factors (for example, ASCL2) and subtype-specific regulators linked to iCMS programs, are mapped along this trajectory to illustrate how chromatin accessibility reshapes cellular identity during CRC progression. *CGI* CpG Island, *ecDNA* extrachromosomal DNA, *SNV* Single nucleotide variation.

Single-cell epigenomic profiling has revealed that CRC heterogeneity is fundamentally encoded at the level of regulatory landscape (Fig. 2a). Rather than presenting a uniform disease state with transcriptional variation, CRC comprises distinct regulatory states defined by divergent *cis*-regulatory element (CRE) landscapes and transcription factors^27^.

Although multiple CRC classification models have been proposed, the most widely used classification method is consensus molecular subtype (CMS) based on bulk transcriptome^59^. This subtyping stratifies CRC into four groups (CMS1–4) and is well correlated with clinical features, including patient prognosis. However, a large-scale scRNA-seq study showed that CMS subtyping inevitably included admixture of diverse cell types, obscuring the intrinsic transcriptomic features of tumor cells. Thus, by leveraging single-cell transcriptomics, a novel subtyping framework based on isolated tumor cells, termed intrinsic CMS (iCMS), was proposed^56^.

Profiling the tumor cell-intrinsic transcriptome reclassified CMS groups: CMS2 as iCMS2, CMS1 and CMS3 as iCMS3, while the fibrotic CMS4 group comprised cancers with either iCMS2 or iCMS3 epithelial cells, indicating that fibrosis is orthogonal to the intrinsic CRC epithelial dichotomy. Furthermore, while iCMS2 subtype primarily arises in the left-sided colon, iCMS3 subtype mainly arises in the right-sided colon^56^, suggesting that their dichotomy may stem from differences in embryonic endodermal origin^60^.

Recently, a large-scale snATAC-seq analysis also uncovered two epigenomic states corresponding to iCMS2 and iCMS3 molecular classifications^27^, suggesting that this transcriptomic dichotomy may also be reflected at the chromatin level (Fig. 1b). iCMS2 tumors maintain accessibility at CREs exhibiting footprinting signatures of intestinal lineage-defining transcription factors, including HNF4A, CDX2, PPARA and ASCL2^27^. These regulatory elements control genes involved in intestinal differentiation, lipid metabolism and canonical Wnt-driven stem cell renewal^61,62^. The retention of this ‘corrupted intestinal’ regulatory program suggests that iCMS2 tumors co-opt normal intestinal stem cell networks for malignant purposes. By contrast, iCMS3 tumors exhibit a dedifferentiated regulatory landscape characterized by accessibility at stress-responsive and mesenchymal enhancers. These regions are enriched for AP-1 (FOS/JUN), TEAD, SOX family and MAFK binding motifs often involved with transcription factors associated with inflammatory signaling, mechanotransduction and epithelial–mesenchymal plasticity^27,32^. The iCMS3 state reflects active disengagement from intestinal lineage programs and adoption of a more primitive, stress-tolerant regulatory configuration. Nonetheless, validation of this dichotomy across independent CRC patient cohorts using snATAC-seq, spanning different stages of tumor progression, will be necessary to establish chromatin-based subtyping as a robust framework.

The intrinsic regulatory heterogeneity provides a mechanistic explanation for why genetically similar CRC tumors exhibit profoundly different clinical behaviors. Patient-derived organoids faithfully recapitulate the tumor-specific regulatory landscapes through extended culture^25^, implying the robustness of tumor-intrinsic regulatory characteristics. Moreover, deep learning analyses trained on pan-cancer snATAC-seq datasets show that chromatin accessibility patterns alone can predict tumor lineage, differentiation state and oncogenic dependencies with high accuracy^22,23^, indicating that CRC regulatory landscapes encode a cellular identity ‘fingerprint’. Taken together, the regulatory architecture defined by snATAC-seq provides a core, mechanistic and clinically relevant framework for understanding tumor identity, one that integrates genetic alterations, microenvironmental influences and cellular plasticity into a unified epigenomic model.

### Dynamic regulatory rewiring drives malignant progression

CRC progression involves extensive regulatory rewiring that reshapes the landscapes of chromatin accessibility beyond original cellular identity (Fig. 2b). Single-cell epigenomic profiling across the adenoma–carcinoma sequence has revealed that malignant transformation proceeds through stepwise reconfiguration of enhancer landscapes, transcription factor networks and lineage programs^24,27^.

#### Loss of intestinal identity during early transformation

The earliest detectable epigenetic changes occur during the transition from normal mucosa to adenomatous polyps. This early stage is marked by the progressive loss of accessibility at enhancers enriched for intestinal differentiation factor motifs, including GATA4/6, KLF4/5 and HOX family members^24,27^. These transcription factors normally maintain the differentiated columnar epithelial phenotype and regulate absorptive and secretory lineage commitment^63,64^. Their silencing may represent an initial step toward dedifferentiation and acquisition of proliferative capacity.

Concomitantly, distal CREs associated with Wnt/β-catenin signaling, a well-established driver of colorectal tumor initiation, gain accessibility. These CREs include regulatory elements targeting *MYC* and its downstream target genes^27^. Epigenetic activation of Wnt-responsive CREs in adenoma-resident intestinal stem cells, which does not occur in isolated *Apc*-null stem cells in a mouse model, suggests that chromatin remodeling associated with mutant crypt interactions is an early event in colorectal tumorigenesis^65^. This finding challenges the traditional linear genetic model of CRC development and instead highlights epigenetic plasticity as a potential initiating layer in tumorigenesis.

#### Regulatory bifurcation through the adenoma–carcinoma transition

The most extensive chromatin remodeling occurs during the transition from late adenoma to invasive carcinoma, a stage that coincides with acquisition of metastatic potential and therapeutic resistance. At this critical junction, tumors commit to one of two dominant epigenetic regulatory trajectories corresponding to iCMS2 or iCMS3 states^27^. For tumors adopting the iCMS2 state, regained chromatin accessibility at intestinal transcription factor loci, including hepatocyte nuclear factor 4 alpha (HNF4A), has been observed^27^. Notably, longitudinal profiling across tumor progression stages revealed that HNF4A accessibility decreases during early adenoma formation but becomes elevated again in established CRC, suggesting a stage-dependent regulatory shift^24^. Such regulatory rewiring may enable tumor cells to exploit altered metabolic programs while maintaining a partially differentiated state.

By contrast, tumors following the iCMS3 state undergo more radical dedifferentiation, as initially defined at the transcriptomic level^56^. At the epigenetic level, this CRC state exhibits widespread accessibility at CREs enriched for AP-1 motifs compared with iCMS2^27^. This observational pattern aligns well with a recent mechanistic study using human CRC organoids, which suggests that AP-1 activation plays a key role in driving dedifferentiation away from colonic epithelial identity toward a fetal state by suppressing intestinal lineage transcription factors such as CDX2 and HNF4A^66^. Interestingly, these identical lineage transcription factors maintain robust accessibility in the iCMS2 state^27^, highlighting how reciprocal chromatin accessibility profiles of these master regulators correlate with distinct CRC phenotypes. In addition, NF-κB-mediated gene expression program was upregulated in the transcriptome profile of iCMS3^56^. Previously, a mouse model study suggested that the activation of NF-κB alone was sufficient to induce dedifferentiation of intestinal epithelial cells and initiate tumorigenesis^32^. Given its potent capacity for cellular reprogramming in the mouse model system, NF-κB signaling may contribute to the inflammatory regulatory state of iCMS3 in human CRC.

#### Oncofetal reprogramming as an emerging regulatory axis in CRC

The concept of ‘oncofetal reprogramming’, the reactivation of fetal-like gene programs in cancer, has recently garnered attention for its role in metastasis and treatment resistance. During normal intestinal development, fetal progenitor cells express a distinct set of transcription factors and utilize CREs that are subsequently silenced after birth. In CRC, these developmental CREs become reactivated, enabling tumor cells to access invasive and migratory programs normally restricted to embryonic contexts. Studies on LGR5-based cell states in CRC have provided key insights into phenotypic plasticity between stem and oncofetal identity.

*LGR5* expression has emerged as a key marker distinguishing tumor cell populations in CRC. Early studies established that *LGR5*^+^ cancer cells function as cancer stem cells, yet their ablation failed to induce tumor regression, as *LGR5*^−^ cells could reconstitute the *LGR5*^+^ population^67,68^. Subsequently, a lineage-tracing experiment demonstrated that *LGR5*^−^ cells, rather than *LGR5*^+^ cells, preferentially migrate to metastatic sites and transition back to *LGR5*^+^ cells to proliferate^69^. Together, these findings raise the question of what distinguishes the *LGR5*^−^ population from its *LGR5*^+^ counterpart.

Multiple independent studies have reported reactivation of fetal gene programs in *LGR5*^−^ tumor cells during CRC tumorigenesis, collectively suggesting that this population undergoes oncofetal reprogramming. Initially, oncofetal reprogramming mediated by YAP signaling was characterized in a mouse model recapitulating CMS1, which is highly overlapping with iCMS3^70^. A subsequent snATAC-seq study in mouse tumoroids resembling the human iCMS2 state extended this finding, suggesting that oncofetal reprogramming may represent a universal program across CRC states. Notably, treatment with FOLFIRI upregulated oncofetal gene expression in these tumoroids, and oncofetal cells showed reduced sensitivity to the treatment, suggesting that oncofetal reprogramming may contribute to acquired chemoresistance^36^. Spatial transcriptomic analysis of human tissue further revealed that oncofetal cells emerge at the invasive front of early CRC, coinciding with decreased *LGR5* expression^7^. Taken together, these studies suggest that CRC cells exist in two phenotypic states, *LGR5*^+^ stem and *LGR5*^−^ oncofetal, and that the transition between them underlies key aspects of metastasis and chemoresistance.

While the precise timing of oncofetal reprogramming remains unclear, two observations offer complementary perspectives. In one observation, aberrant fetal gene expression was detected as early as the adenoma stage in a study using mouse models and patient-derived organoids^35^; however, as this study relied on mouse data and organoids derived exclusively from patients with familial adenomatous polyposis, whether these findings well reflect the sporadic CRC context remains uncertain. By contrast, spatial transcriptomic analysis of human tissue suggests that oncofetal cells emerge coinciding with invasive front formation in early-stage T1 CRC, driven by TGF-β signals from trophocyte-like CAFs^7^. Single-cell epigenomic profiling of human adenoma and early CRC before invasive front formation could bridge these observations and may further clarify how epigenomic rewiring directs the onset of oncofetal reprogramming.

Extensive research has begun to dissect the underlying mechanisms of oncofetal reprogramming, but the exact mechanisms remain only partially elucidated. Mechanistic insights from experimental organoid models suggest that oncofetal reprogramming is regulated at multiple epigenetic levels. At the chromatin level, three-dimensional (3D) genome architecture and promoter accessibility are broadly similar between the adult and fetal intestine; however, enhancer accessibility differs markedly^34^. Supporting the importance of chromatin accessibility in fetal-like states, mouse normal intestinal organoids in which fetal-like reversion was induced by TGF-β showed elevated chromatin accessibility with enriched SOX, TEAD and SMAD family motifs^71^. In the tumor context, a mouse tumoroid study using snATAC-seq further suggested that the *LGR5*^+^ stem state is enriched by ASCL2 and TCF-LEF family motifs, whereas the *LGR5*^−^ oncofetal state is preferentially enriched by YAP/TAZ-TEAD motifs^36^ (Fig. 1c). At the histone-modification level, LSD1-controlled H3K4me1/2 represses fetal-like gene programs at enhancers^72^, while PRC2-mediated H3K27me3 acts at broader regulatory regions including gene promoters^73^. Although these histone modification studies were conducted in nontumorigenic model systems under perturbation conditions, oncofetal reprogramming in human CRC may similarly be driven by disrupted chromatin accessibility and histone modifications, collectively creating a permissive epigenetic environment for stochastic gene expression. These epigenetic changes may contribute to stochastic enhancer–promoter interactions^74^ that are nonlinearly translated into transcriptional bursting dynamics at the single-cell level^75^, creating a pool of phenotypic diversity. Such a plastic phenotypic landscape of cancer cells may act as a distinct mechanism of nongenetic evolution that enables rapid adaptation without the requirement for de novo genetic mutations^8,76^.

Collectively, these lines of evidence raise two key unresolved questions. First, two independent experimental models, including an in vivo mouse model and a mouse tumoroid study, implicate YAP/TAZ signaling as a core regulatory factor in oncofetal reprogramming, but it is unclear whether a similar mechanism may operate across human CRC states regardless of their regulatory status. Second, whether chemotherapy selects for pre-existing oncofetal cells or actively induces this state through the same YAP/TAZ activation remains to be determined. Should YAP/TAZ prove to be a shared mechanism, it would be intriguing that iCMS2 and iCMS3, despite their fundamentally divergent chromatin landscapes, could converge toward shared metastatic and therapy-resistant phenotypes through a common regulatory axis, potentially positioning YAP/TAZ signaling as a subtype-agnostic therapeutic vulnerability.

#### Hepatotropic regulatory remodeling in colorectal cancer metastasis

The ultimate manifestation of regulatory plasticity is metastatic colonization, which requires tumor cells to survive and proliferate in new tissue environments. snATAC-seq profiling of matched primary colon tumors and liver metastases has revealed organotropic chromatin remodeling in mouse models^30^. During liver colonization, metastatic cells progressively lose accessibility at colon-specific enhancers (enriched for IRF1, ELF1 and STAT1 motifs) while gaining accessibility at liver-specific regulatory elements (enriched for HNF4A, FOXA2 and CTCF motifs)^30^. This epigenetic transition generates hybrid cellular states that express both epithelial and hepatocyte-like gene programs. The acquisition of HNF4A-driven CRE landscapes in liver metastases is reminiscent of the iCMS2 regulatory signature in CRC^27^. Whether primary tumors with iCMS2 characteristics may be selectively advantaged for hepatic colonization remains an intriguing possibility, consistent with the clinical observation that left-sided (iCMS2-enriched) CRCs preferentially metastasize to liver^77^.

Importantly, chromatin remodeling during metastasis is accompanied by shifts in enhancer–promoter connectivity. Co-accessibility analysis suggests an expansion of long-range regulatory contacts favoring liver-specific circuits, particularly around *HNF4A* and *FOXA2* loci^30^. This rewiring may enable metastatic cells to respond to liver-specific growth factors and metabolic signals, facilitating adaptation to the hepatic niche.

#### An integrative temporal model of regulatory evolution

Collectively, these observations support a temporal model in which CRC progression is driven by sequential waves of regulatory rewiring: (1) initial loss of differentiation programs and activation of Wnt signaling in adenomas; (2) regulatory bifurcation into iCMS2 or iCMS3 states during malignant transformation; (3) activation of oncofetal plasticity programs that enable therapy resistance; and (4) organotropic regulatory adaptation during metastasis. Each transition involves coordinated changes across thousands of enhancers, creating new transcriptional possibilities while constraining others. This regulatory evolution operates in concert with the genetic background during tumorigenesis, suggesting an epigenomic layer that enables even genetically identical tumor cells to exhibit such profound phenotypic diversity^9,16^.

### Microenvironmental co-evolution through epigenetic crosstalk

The regulatory rewiring of tumors is not a unique property of malignant epithelial cells but extends to stromal and immune compartments. Single-cell epigenomic profiling has revealed that stromal and immune populations within the TME also undergo dynamic chromatin remodeling that may reciprocally interact with the regulatory states of cancer cells^24^ (Fig. 1c). Furthermore, similar to malignant cells, a recent study suggests that tumor-associated endothelial cells also co-opt their developmental programs for tumor angiogenesis governed by epigenetic plasticity^78^. While single-cell transcriptomic and functional studies in model systems have begun to illuminate malignant cell–TME communications^79–81^, how these interactions are encoded at the epigenomic level in CRC remains largely unexplored. We therefore synthesize current knowledge of regulatory rewiring in the TME on the basis of chromatin accessibility and suggest the concept of an iCMS-defined epigenomic ecosystem as a framework for future investigation.

#### Regulatory diversification in response to tumor signals through CAFs

Normal tissue fibroblasts maintain relatively uniform chromatin accessibility profiles enriched for homeostatic programs. On exposure to tumor-derived signals, these cells undergo dramatic epigenetic reprogramming that generates functionally distinct CAF subtypes with opposing roles in tumor biology^25,38,39^. Tumor-promoting CAFs exhibit chromatin accessibility enriched for RUNX1, AP-1, NF-κB and HIF1A motifs, which are transcription factors activated by inflammatory cytokines, hypoxia and growth factors secreted by tumor cells^24,38^. These regulatory elements control genes involved in extracellular matrix remodeling, angiogenesis and immunosuppressive cytokine production.

By contrast, tumor-restraining CAFs (Tr-CAFs) retain accessibility at enhancers associated with normal tissue architecture and extracellular matrix homeostasis^39^. These cells express collagens, laminins and basement membrane components that may physically constrain tumor expansion. Tr-CAFs are progressively depleted during malignant transformation^39^, suggesting active selection against their tumor-restraining functions.

The regulatory divergence between tumor-promoting CAFs and Tr-CAFs is not genetically determined but rather reflects epigenetic responses to distinct microenvironmental niches. This is indicated by various experiments showing that normal fibroblasts acquire CAF-like histone modification patterns when exposed to tumor-conditioned media^82^. Furthermore, recent findings highlight that cancer cell-intrinsic epigenetic remodeling, such as the rewiring of H3K36me3-mediated regulatory programs, can actively shape the characteristics and abundance of CAFs^83^. Given that the spatial niches of CAFs are well conserved across various cancer types^84^, the functional heterogeneity of CAFs appears to be orchestrated by their neighboring compartments via these diverse epigenetic mechanisms.

#### Progressive establishment of immunosuppression via T cell exhaustion and loss of stemness

The immune compartment undergoes equally profound epigenetic remodeling during CRC progression, with chromatin accessibility changes that progressively favor immunosuppressive over cytotoxic functions^11,24,40^. CD8^+^ T cells in CRC exhibit progressive loss of accessibility at the *TCF7* locus, a master regulator of T cell stemness and proliferative capacity^11^. Mechanistically, *Tcf7*-deficient mouse models have revealed that this factor acts as an essential pioneer factor required to open repressed chromatin domains^41^. Consequently, the epigenetic silencing of *TCF7* in the CRC TME may mark the transition to terminal exhaustion, characterized by chromatin accessibility enriched for SOX10, MEIS1 and RORα^11^. Consistent with these findings, a longitudinal study has also revealed that this T cell exhaustion and loss of stemness is already partially established in adenomas and becomes complete in carcinomas^24^. The early initiation of T cell exhaustion suggests that immune suppression is not merely a consequence of advanced disease but rather an enabling event that permits malignant transformation, consistent with the concept of immunosuppressive niche engineering as a bottleneck in tumor evolution^40^.

#### T_reg_ heterogeneity

Regulatory T cells (T_regs_) expand progressively from normal tissue through adenoma to carcinoma, but snATAC-seq has revealed that this population is epigenetically heterogeneous^43^. Two distinct T_reg_ subtypes can be distinguished by their chromatin accessibility profiles. An snATAC-seq study from mouse tumoroids resembling human microsatellite-stable CRC has revealed IL-10^+^ T_reg_ cells enriched for RORC motifs and IL-10^−^ T_reg _ cells enriched for GATA and NR4A family motifs^43^, with distinct functional consequences. Beyond these observational profiles, subsequent functional experiments revealed their respective roles. Building on previous work^44^, functional analyses suggest that IL-10^−^ T_reg_ cells displaying NR4A motifs exhibit stronger T cell receptor (TCR) signaling and more potent immunosuppressive activity. By contrast, IL-10^+^ T_reg_ cells suppress tumor growth by hindering IL-17 production^43^. Clinically, a high density of IL-10^+^ T_reg_ cells correlates with favorable prognosis, whereas an abundance of IL-10^−^ T_reg_ cells predicts poor outcomes^43^. The observation of two distinct T_reg_ subtypes may resolve the longstanding paradox where T_reg_ infiltration is associated with good prognosis in CRC but poor prognosis in most other solid tumors^42^. Taken together, these findings suggest the prognostic impact depends on the epigenetically defined T_reg_ subtype, not simply T_reg_ abundance.

#### Macrophage polarization

A single-cell transcriptome analysis revealed that tumor-associated macrophages (TAMs) in CRC exhibit a dichotomous distribution into C1QC^+^ and SPP1^+^ subtypes, unlike the continuous polarization spectrum observed in breast and lung cancers^37^. snATAC-seq profiling reveals that these subtypes possess distinct regulatory architectures^11^. C1QC^+^ TAMs are enriched for ETV family, ELK4, ZBTB7A, YY1 and RUNX3 motifs and localize primarily in adjacent normal and precancerous tissues, where they maintain tissue homeostasis and support antitumor immunity^45^. SPP1^+^ TAMs, by contrast, exhibit accessibility at KLF14 and KLF16 motifs and accumulate within tumor tissues^11^. The distinct chromatin accessibility profiles between C1QC^+^ and SPP1^+^ TAMs suggest that divergent regulatory programs may underlie their opposing functional roles in the TME.

Collectively, these observations suggest that CRC regulatory landscapes reflect a coordinated epigenomic ecosystem, defined here as an integrated epigenomic state shaped by crosstalk across malignant, stromal and immune compartments, rather than cell-autonomous states. Notably, TME composition varies with iCMS states; for example, iCMS3 exhibited elevated fibroblast, endothelial and immune cell transcriptomic signatures compared with iCMS2^56^. Furthermore, the iCMS3-specific chromatin peaks in malignant cells revealed upregulation of diverse immune pathways including leukocyte activation^27^, implying that crosstalk between TME and malignant cells may be tuned at the epigenomic level. Understanding how these compartment-specific epigenomic programs are coordinated, and whether iCMS identity instructs or is reinforced by microenvironmental remodeling, will be essential to establish the regulatory basis of iCMS-defined epigenomic ecosystems.

### Genomic–epigenomic crosstalk in reshaping the epigenetic landscape

While chromatin accessibility profiling reveals extensive nongenetic heterogeneity in CRC, the regulatory landscape itself is profoundly shaped by genomic alterations (Fig. 1c). Rather than acting independently, genetic and epigenetic mechanisms engage in bidirectional crosstalk: mutations alter chromatin accessibility, while chromatin states influence mutational processes and selection pressures. Single-cell epigenomic approaches integrated with genomic analyses have begun to illuminate this complex interface^47,85^.

#### Noncoding mutations rewire transcription factor binding

The majority of somatic mutations in cancer occur in noncoding regions, yet their functional consequences have remained largely obscure. snATAC-seq provides a direct readout of how noncoding mutations affect regulatory element activity by measuring changes in chromatin accessibility at mutated loci. Systematic analysis of CRC genomes has identified recurrent noncoding mutations in regulatory sequences, particularly within CREs^48,49^.

A notable example is the *ETS2* enhancer, which harbors recurrent mutations that alter ETS family transcription factor binding and increase *ETS2* expression, a change that promotes epithelial–mesenchymal transition and metastatic potential^48^. Similarly, mutations in the *TERT* promoter create de novo ETS binding sites, leading to telomerase reactivation and unlimited replicative potential^49^. Integration of bulk ATAC-seq with whole-genome sequencing across large CRC cohorts has revealed that regulatory mutations frequently occur in accessible chromatin regions and correlate with expression changes in nearby genes^49^.

Recent application of deep learning models trained on single-cell chromatin accessibility data has enabled the prediction of how individual noncoding mutations alter accessibility in specific cellular contexts^22^. These models reveal that the same mutation can have opposing effects in different cell types, increasing accessibility in malignant cells while decreasing it in stromal cells. Moreover, snATAC-seq has uncovered that certain regulatory mutations create ‘neo-enhancers’ that rewire transcriptional networks^86^. These mutations do not simply modulate existing regulatory elements but rather generate entirely new binding sites for transcription factors that would not normally regulate the target gene. This mechanism represents a form of regulatory innovation that enables tumor cells to escape normal developmental constraints.

#### 3D genome reorganization and enhancer hijacking

Beyond point mutations, large-scale structural variations including translocations, deletions, inversions and complex rearrangements profoundly reshape the 3D architecture of the cancer genome^50^. These topological changes can bring oncogenes into contact with distant super-enhancers, a phenomenon termed ‘enhancer hijacking’. Spatial 3D genome mapping integrated with H3K27ac chromatin immunoprecipitation followed by sequencing (ChIP-seq) in CRC has revealed widespread enhancer hijacking events, particularly affecting *MYC* and *CCND1* oncogenes^47^. In normal cells, these genes are insulated from nearby super-enhancers by CTCF-bound boundary elements that organize the genome into discrete topologically associating domains. Structural variations that reorganize topologically associating domains enable aberrant long-range contacts between oncogene promoters and super-enhancers that normally regulate unrelated genes.

Critically, an snATAC-seq study in another cancer type has suggested that hijacked enhancers retain their native chromatin accessibility and activity states^87^, raising the possibility that similar mechanisms may operate in CRC. These enhancers are not simply ‘turned on’ by the rearrangement but rather redirect their pre-existing regulatory output to new target genes^47^. Such a mechanism may explain why enhancer-hijacking events are oncogenic: they co-opt highly active, cell-type-appropriate regulatory elements to drive inappropriate oncogene expression. Meanwhile, the concept of ‘active support’ has emerged from the large-scale analysis of enhancer hijacking^47^. Cancer-specific 3D genome reorganization preferentially activates genes with known oncogenic functions in a nonrandom manner, suggesting that only those rearrangements that activate growth-promoting or survival-enhancing genes may be retained during tumor evolution.

#### Extrachromosomal DNA as mobile regulatory platforms

Extrachromosomal DNA (ecDNA) represents an extreme form of genomic alteration that profoundly impacts the epigenetic landscape^46,51,52^. These circular DNA elements, which arise from chromothripsis or breakage-fusion-bridge cycles, carry amplified oncogenes along with their associated enhancers, creating autonomous regulatory units that exist outside the constraints of chromosomal topology.

**Fig. 3:**
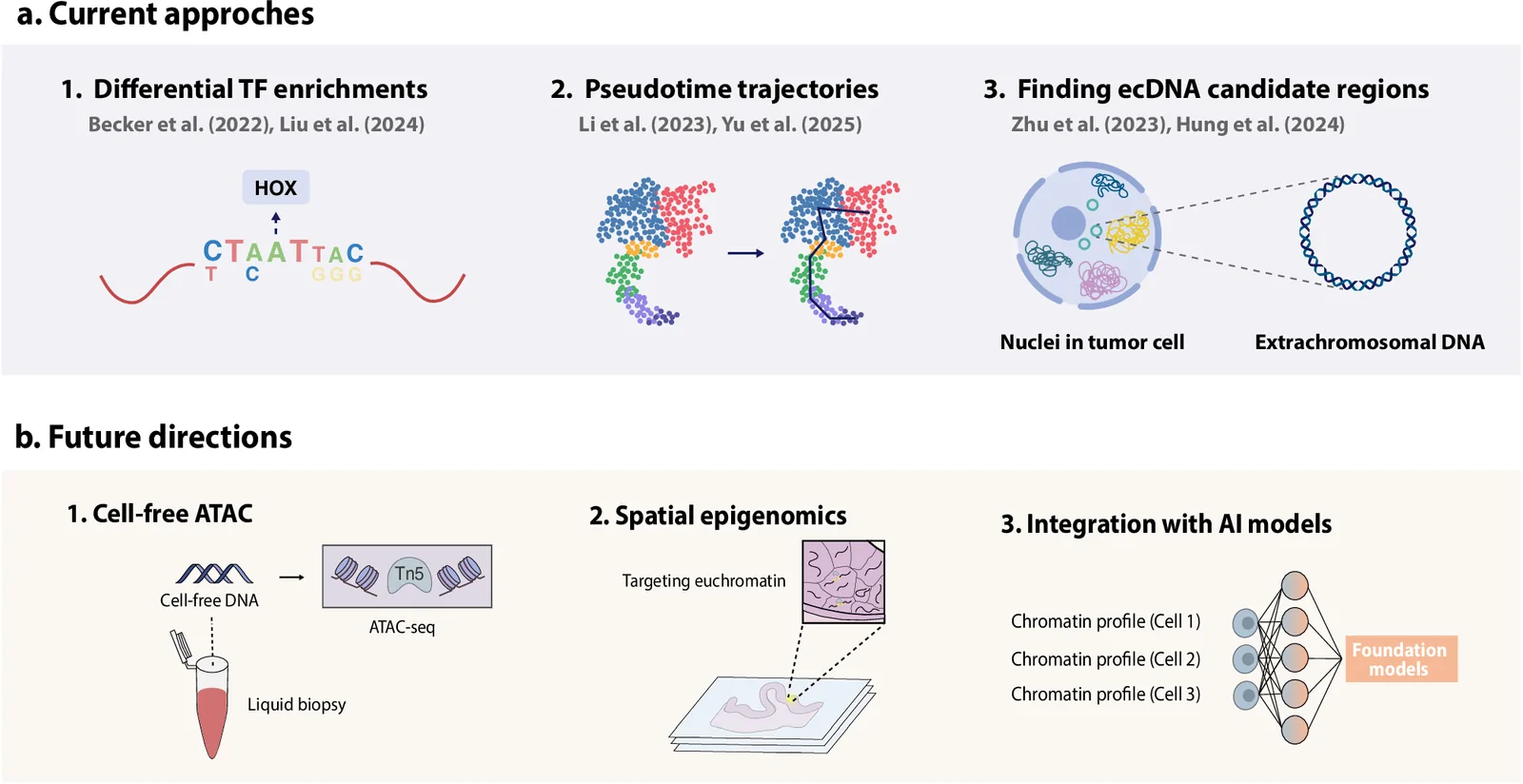
Current and future applications of snATAC-seq for dissecting epigenetic characteristics of cancer. **a** Current applications of snATAC-seq in (1) profiling differentially accessible regulatory elements and transcription factor (TF) motif enrichment to infer regulatory programs, (2) reconstructing malignant epigenetic trajectories using pseudotime analysis and (3) identifying ecDNA through aberrant chromatin accessibility patterns. **b** Future directions include (1) extending chromatin profiling to liquid biopsy-based assays, (2) incorporating spatial epigenomic information and (3) integrating these datasets with AI-based predictive models to enhance disease monitoring and therapeutic decision-making.

snATAC-seq has been instrumental in identifying ecDNA-bearing cells within heterogeneous tumor populations (Fig. 3a). Simultaneous analysis of copy number variation and chromatin accessibility in a single cell suggested that ecDNA harboring oncogenes confers a growth advantage on cells in many cancer cell lines, including CRC^46^. This increased accessibility may contribute to elevated transcriptional output of oncogenes located on ecDNA, potentially explaining the selective advantage and cellular heterogeneity conferred by ecDNA-bearing cells.

Recent single-cell studies have addressed a longstanding question about ecDNA inheritance: how are these extrachromosomal elements distributed during cell division? Time-lapse imaging combined with single-cell genomics has revealed that ecDNA copies undergo coordinated segregation during mitosis, with different ecDNA species exhibiting co-selection and co-inheritance in cancer cell lines including CRC^52^. The nonrandom segregation ensures that daughter cells receive functional combinations of ecDNA elements, maintaining oncogenic regulatory networks across cell divisions.

The epigenetic heterogeneity generated by ecDNA also has direct clinical relevance. ecDNA copy number varies dramatically between individual tumor cells, creating subpopulations with different levels of oncogene expression and drug sensitivity^88^. Cells with a high ecDNA copy number may be more sensitive to targeted therapies but also more capable of rapid adaptation through ecDNA loss. Conversely, cells with a low ecDNA copy number may survive initial treatment and subsequently re-amplify ecDNA, driving resistance.

#### Genomic–epigenomic feedback loops

These findings establish that genomic alterations and epigenetic states exist in dynamic equilibrium. Mutations create new regulatory possibilities by altering transcription factor binding sites, structural variations rewire enhancer–promoter contacts and ecDNA generates mobile regulatory platforms, all of which reshape chromatin accessibility landscapes. Reciprocally, chromatin states influence which genomic regions are accessible to mutagenic processes and which alterations provide selective advantages.

This genomic–epigenomic crosstalk explains several puzzling features of CRC biology. First, it accounts for why tumors with similar mutational profiles can exhibit vastly different phenotypes. The same genetic alterations produce different outcomes depending on the epigenetic context in which they occur^9,16^. Second, it explains the rapid adaptation observed in response to therapy. While acquiring new driver mutations is slow, epigenetic remodeling of existing genomic alterations can quickly generate resistant phenotypes^8,76^. Beyond these observations, genomic–epigenomic crosstalk may also explain why certain genomic alterations are preferentially selected in specific contexts, such as CRC states. Recent genomic studies on APC driver mutations have suggested that the selection of specific mutation sites is not solely determined by the mutation itself but is shaped by the surrounding environment that modulates the optimal threshold of oncogenic signaling required for transformation^89–91^. For instance, the co-occurrence of *KRAS* and *APC* mutations results in distinct APC truncation patterns compared with*APC* mutation alone, favoring mutations that reduce WNT signaling^91^. These findings suggest that the genomic context shaped by one driver mutation can influence the selective pressure on another. Building on this concept, further investigation into how driver mutations shape the epigenome in CRC, as shown in other tumors such as pancreatic ductal adenocarcinoma^92^ and breast cancer^93^, and conversely how such epigenomic changes influence which genomic alterations confer selective advantage in specific contexts, will be essential for a deeper understanding of the ‘permissive field’ concept.

### The outlook toward translational and therapeutic applications

The mechanistic insights gained from single-cell epigenomic profiling of CRC are now being translated into clinical applications and next-generation research strategies. These advances span therapeutic targeting, noninvasive monitoring, spatial contextualization and AI-driven modeling, collectively promising to transform precision oncology for CRC (Fig. 1d,e).

#### Targeting the epigenetic–immune interface

The revelation that chromatin states directly regulate immune evasion programs has opened new therapeutic avenues combining epigenetic modulators with immunotherapy^53^. CIMP-high CRCs, which exhibit widespread promoter hypermethylation and H3K27me3-mediated silencing of immune gene enhancers, are typically resistant to checkpoint blockade. However, treatment with DNA methyltransferase inhibitors (DNMTi) or EZH2 inhibitors can reactivate these silenced enhancers, restoring antigen presentation and chemokine expression. Early-phase clinical trials combining low-dose DNMTi with anti-PD-1 antibodies have shown promising response rates in microsatellite-stable CRC, a population that typically does not respond to immunotherapy alone^53^. snATAC-seq profiling of pre- and post-treatment biopsies reveals that responders exhibit increased accessibility at interferon-responsive enhancers and MHC class I regulatory elements, providing a mechanistic biomarker for treatment efficacy.

Beyond DNA methylation, targeting enhancer-associated regulators represents an emerging strategy (Fig. 1e). BET bromodomain inhibitors are known to displace transcriptional co-activators from super-enhancers. Given that combinatorial therapeutic strategies—pairing BET bromodomain inhibitors with Wnt (upregulated in iCMS2) or MAPK (upregulated in iCMS3) inhibitors—have shown preclinical efficacy in CRC cell lines^94^, cotargeting super-enhancers with subtype-specific regulatory drivers offers a promising approach for precision CRC therapy.

Notably, studies in mouse models have demonstrated that specific compounds can reshape chromatin accessibility to enhance antitumor immunity through distinct mechanisms. Arenobufagin increases accessibility at HSP90β regulatory elements, disrupting the HSP90β–STAT3–PD-L1 axis and sensitizing tumors to T cell-mediated killing^28^. Cycloastragenol enhances accessibility at antigen presentation genes through cathepsin-B inhibition, promoting CD8^+^ T cell infiltration and synergizing with PD-1 blockade^29^. These examples illustrate how pharmacological interventions can modulate tumor–immune crosstalk through chromatin remodeling, providing rational combinations for clinical development.

#### Metabolic-epigenetic coupling as a therapeutic vulnerability

An unexpected finding from single-cell epigenomic studies is the tight coupling between cellular metabolism and chromatin states^95^. Fe3^+^-dependent telomerase reactivation, mediated through iron-responsive transcription factors that bind telomerase regulatory elements, exemplifies how metabolic states directly influence chromatin accessibility^26^. This discovery suggests that metabolic interventions, such as iron chelation or modulation of redox balance, may indirectly reprogram the epigenome. More broadly, the observation that iCMS2 tumors exhibit preferential HNF4A accessibility suggests vulnerability to metabolic inhibitors targeting fatty acid oxidation or lipid synthesis^61,62^ (Fig. 1e). Conversely, iCMS3 tumors show transcriptomic enrichment of glycolysis pathways^56^ and chromatin accessibility at NRF2 motifs^27^, suggesting sensitivity to inhibitors of glucose metabolism or antioxidant pathways^96^. Integrating snATAC-seq with metabolomic profiling in patient-derived organoids could enable prediction of metabolic dependencies based on regulatory architecture.

#### Liquid biopsy as a tool for noninvasive epigenomic monitoring

Circulating cell-free DNA (cfDNA) released from tumors carries epigenetic information, including DNA methylation patterns and nucleosome positioning that reflects chromatin accessibility. While cfDNA mutation profiling has been widely developed for cancer diagnosis, achieving high specificity in early-stage detection remains challenging owing to the presence of mutations in cancer-associated genes within healthy individuals^97^. The recent development of cell-free ATAC-seq enables profiling of open chromatin landscapes from blood samples (Fig. 3b). Proof-of-concept studies in breast and pancreatic cancer have demonstrated that cfDNA enrichment at cancer-specific open chromatin regions can distinguish patients with cancer from healthy donors and reflect immune microenvironment changes^54^. Extending this approach to CRC may offer similar clinical utility, particularly given the epigenomic heterogeneity characteristic of this disease. Importantly, cell-free ATAC-seq captures the global regulatory state of tumors rather than individual mutations, potentially providing earlier detection of resistance mechanisms that arise through epigenetic rather than genetic changes. Furthermore, a cf-chromatin state with XGBoost classifier has been proposed for predicting disease origin and its prognosis in CRC^98^. Building on this, machine learning models trained on snATAC-seq data from tumor tissues present a promising framework for enhancing the sensitivity of liquid biopsy. This could potentially enable longitudinal monitoring of epigenetic evolution during therapy, early identification of resistance and the selection of patients likely to benefit from epigenetic-targeted interventions.

#### Spatial epigenomics for contextualizing regulatory states

While single-cell epigenomics has resolved cellular heterogeneity, it typically requires tissue dissociation that destroys spatial information. Emerging spatial epigenomic technologies, including Slide-ATAC and spatial multi-ome approaches, now enable in situ profiling of chromatin accessibility while preserving tissue architecture^55^ (Fig. 3b). Application of these methods to CRC has revealed that regulatory states are not randomly distributed but rather organized into spatial domains^55,56^.

For example, cells with oncofetal chromatin signatures localize to the tumor–stroma interface, where they encounter mechanical stress and inflammatory signals that maintain plasticity programs. Exhausted T cells cluster in regions distant from functional vasculature, suggesting that hypoxia contributes to their epigenetic silencing of effector programs.

Spatial epigenomics also reveals how anatomical location within the colon influences regulatory architecture. Right-sided and left-sided CRCs exhibit distinct chromatin accessibility patterns that reflect their developmental origins from different segments of the embryonic gut^56,57^. These location-specific regulatory programs influence not only tumor biology but also patterns of metastatic spread with left-sided tumors, exhibiting preprogrammed hepatic enhancer signatures that facilitate liver colonization^30,77^.

#### Application of AI for modeling regulatory logic

The complexity and scale of single-cell epigenomic data, often comprising millions of cells and hundreds of thousands of regulatory elements, necessitates advanced computational approaches. AI, particularly deep learning, has emerged as an indispensable tool for extracting regulatory logic from these datasets^22,23,58^ (Fig. 3b). Graph neural networks that model enhancer–promoter interactions as network structures can predict gene expression from chromatin accessibility with remarkable accuracy. These models learn the combinatorial rules governing transcription factor cooperation, enabling inference of regulatory networks directly from snATAC-seq data without requiring prior knowledge of TF binding sites^23^. Transformer-based architectures, inspired by large language models, have been trained on massive single-cell epigenome datasets to create ‘regulatory foundation models’^58^. These models encode generalized representations of chromatin states that transfer across cell types and disease contexts. When fine-tuned on CRC data, such models may enable prediction of the effects of regulatory perturbations, simulation of transitions between iCMS2 and iCMS3 states, and nomination of candidate transcription factors whose manipulation could reverse oncogenic programs. Such explorations may ultimately shed light on the precise regulatory triggers underlying iCMS2 and iCMS3 bifurcation, a key open question in the field.

Variational autoencoders trained on integrated multi-ome data (simultaneous chromatin accessibility and gene expression) can generate synthetic cells with desired regulatory properties, enabling virtual screening of epigenetic drug combinations. These models predict which enhancers must be modulated to achieve specific transcriptional outcomes, providing rational design principles for epigenetic therapies. Perhaps most powerfully, AI models can integrate snATAC-seq data with clinical outcomes to identify regulatory signatures predictive of prognosis and treatment response. These signatures often capture features invisible to human interpretation, such as subtle accessibility patterns across thousands of enhancers, that collectively encode therapeutic vulnerability.

### Future directions and remaining challenges

Despite these advances driven by single-cell epigenomic technologies, important challenges remain. Current snATAC-seq protocols capture only a snapshot of chromatin states, missing the temporal dynamics of regulatory remodeling during treatment or metastasis. Emerging technologies enabling longitudinal epigenomic profiling in organoid systems or patient-derived xenografts will be essential for understanding how regulatory states evolve in real time.

Data sparsity inherent to single-cell approaches limits detection of rare cell states and subtle accessibility changes. Improved library preparation methods, deeper sequencing and computational imputation strategies are needed to achieve comprehensive regulatory coverage. Integration with complementary single-cell epigenomic modalities, including single-cell histone modification profiling, DNA methylation mapping and chromosome conformation capture, will provide a more complete view of chromatin regulation^99^. 3D genome organization remains underexplored at single-cell resolution. While bulk Hi-C studies have revealed extensive 3D genome rewiring in CRC^47^, single-cell chromosome conformation technologies are needed to understand how enhancer–promoter contacts vary across cell states and how this variation contributes to regulatory heterogeneity.

Finally, translation of epigenomic insights into clinical practice requires validation in prospective trials. Biomarkers based on chromatin accessibility must demonstrate reproducibility across platforms and institutions, and epigenetic therapies must prove efficacy in randomized studies. The integration of snATAC-seq into clinical workflows from tissue collection to data analysis to therapeutic decision-making will require substantial infrastructure development and regulatory framework evolution.

## Conclusion

Single-cell epigenomic profiling has reframed CRC from a disease of accumulated mutations to one of dysregulated gene regulatory networks. By measuring chromatin accessibility at cellular resolution, these approaches reveal that CRC heterogeneity is primarily encoded in enhancer landscapes, transcription factor networks and 3D genome architecture, which are invisible to genomic and transcriptomic analyses alone.

Three transformative principles emerge from single-cell epigenomics. First, malignant cells in CRC comprise distinct regulatory states (iCMS2 and iCMS3). Second, malignant progression proceeds through stepwise rewiring of the regulatory state. From adenoma differentiation loss, through transformation and oncofetal reprogramming during therapy resistance, to organotropic metastatic adaptation, epigenetic plasticity emerges as the primary driver of nongenetic evolution. Third, genomic alterations and chromatin states exist in bidirectional feedback, explaining why genetically similar tumors exhibit profound phenotypic diversity and why resistance often emerges without new driver mutations.

These insights are now entering clinical translation. Epigenetic–immune combinations exploit chromatin-mediated immunosuppression reversibility. Liquid biopsies enable noninvasive regulatory state monitoring. Spatial epigenomics contextualizes accessibility within tissue architecture. AI trained on single-cell epigenome data can predict regulatory logic and nominate therapeutic vulnerabilities with unprecedented precision. More importantly, given the epigenetic plasticity underlying CRC progression and the recently reported strong association between CRC risk and epigenetic aging^100,101^, a therapeutic strategy capable of ‘reversing this epigenetic clock’—restoring oncofetal or dedifferentiated cells toward mature, lineage-committed regulatory states—may represent a promising future therapeutic direction. Just as normal development proceeds through branching trajectories shaped by environmental cues, signaling gradients and transcriptional programs, therapeutically re-imposing the regulatory determinants of this trajectory in reverse may offer a novel means of constraining tumor plasticity.

Despite such advancement, critical challenges remain. Current technologies capture static snapshots, thus temporal resolution during treatment is needed. Multi-omic data integration remains computationally demanding. 3D genome organization at single-cell resolution represents a missing layer. Translation requires prospective validation and infrastructure development. In the future, the convergence of single-cell multi-omics, spatial technologies and AI-driven modeling promises regulatory-guided precision oncology. By decoding the epigenomic logic governing cellular identity, plasticity and therapeutic response, we gain actionable strategies to disrupt the regulatory programs enabling CRC.
